# *Asparagus cochinchinensis*: A review of its botany, traditional uses, phytochemistry, pharmacology, and applications

**DOI:** 10.3389/fphar.2022.1068858

**Published:** 2022-11-30

**Authors:** Meng Wang, Shuang Wang, Wenjing Hu, Zhibin Wang, Bingyou Yang, Haixue Kuang

**Affiliations:** Key Laboratory of Basic and Application Research of Beiyao (Heilongjiang University of Chinese Medicine), Ministry of Education, Heilongjiang University of Chinese Medicine, Harbin, China

**Keywords:** *Asparagus cochinchinensis* (Lour.) Merr, traditional uses, phytochemistry, pharmacology, applications

## Abstract

*Asparagus cochinchinensis* (Lour.) Merr. (*A. cochinchinensis*) is a traditional herbal medicine that is used to treat constipation, fever, pneumonia, stomachache, tracheitis, rhinitis, cataract, acne, urticaria. More than 90 compounds have been identified from different structural types in *A. cochinchinensis*, including steroidal saponins, C_21_-steroides, lignans, polysaccharides, amino acids, etc. These bioactive ingredients make *A. cochinchinensis* remarkable for its pharmacological effects on anti-asthma, anti-inflammatory, anti-oxidation, anti-tumor, improving Alzheimer’s disease, neuroprotection, gut health-promoting and so on. Moreover, *A. cochinchinensis* also plays an important role in food, health product, cosmetic, and other fields. This review focused on the research publications of *A. cochinchinensis* and aimed to summarize the advances in the botany, traditional uses, phytochemistry, pharmacology, and applications which will provide reference for the further studies and applications of *A. cochinchinensis.*

## Introduction

*A. cochinchinensis* is belonging to the genus *Asparagus* in the family *Liliaceae*, it is widely distributed in temperate and tropical regions, including China, Japan, Korea, and Vietnam. (Kubota et al., 2012; Pegiou et al., 2019; Pahwa et al., 2022). *A. cochinchinensis* is one of the most frequently used traditional herbal medicines, with documented cases of its clinical therapeutic effect in many countries. (Sheng., 2022a; Wong et al., 2022). *A. cochinchinensis* first appeared as a traditional Chinese medicine (TCM) in the earliest Chinese medicinal classic work Shennong’s Classic of Materia Medica (written more than 2000 years ago during the Han Dynasty), it has a long history of medicinal use and its medicinal value has been proved by clinical experience. It was included in the Pharmacopoeia of the People’s Republic of China 1977 edition as a clinical TCM in common use for the first time, and was continuously included until the latest 2020 edition. Dried roots are the main medicinal parts of *A. cochinchinensis*, it has been commonly used either alone or in combination with other herbal medicines to treat asthma, cough, constipation, thrombosis and inflammatory disease in China for centuries. Many classic formulas containing *A. cochinchinensis* have been widely used in clinic and have made important contributions to the health of people in China and other traditional medicinal systems in Asia. In addition to its medicinal value, *A. cochinchinensis* has various commercial applications in health products, food, and cosmetics (Safriani et al., 2022). It is commonly used as a food or nutritional supplement (Siand et al., 2015), cosmetics with whitening and anti-aging effects, and even used as a raw material for fermentation and winemaking (Kim et al., 2017; Topolska et al., 2021). Therefore, its huge potential and broad development prospects are worth exploring.

In the past few decades, *A. cochinchinensis* has attracted widespread attention as an important herbal medicine. Significant progress on isolation and identification of active constituents in *A. cochinchinensis* have been made in relevant researches. So far, more than 90 components have been isolated and identified. They mainly include steroidal saponins, C_21_-steroids, lignans, polysaccharides, and amino acids. At present, *A. cochinchinensis* has a variety of pharmacological effects and has curative effects in the treatment of asthma, tumor, Alzheimer’s disease, gut diseases, inflammatory diseases (Lee et al., 2009; Lei et al., 2016; Choi et al., 2019; Zhang R. S. et al., 2021). Besides that, medicinal prescription research also has revealed that it functions synergistically in combination with various herbal medicines (Weiying et al., 2006; Jung et al., 2014). With the in-depth exploration of TCM the exploitation and utilization of traditional herbal medicine in the prevention and treatment of various diseases are steadily increasing.

With the current scientific and technological advances and the increasing international recognition of traditional herbal medicine in recent years, research on *A. cochinchinensis* has made significant progress. However, to the best of our knowledge, there is no review on *A. cochinchinensis*. It is particularly important and necessary to collate a review on *A. cochinchinensis* progress in recent years. This is the first review on up to date of *A. cochinchinensis* research developments in the fields of botany, traditional uses, phytochemistry, pharmacology, and applications. It provides an accurate overview of *A. cochinchinensis* research and identifies deficiencies in present studies, proposing further research targets. The authors expect this review to encourage further research into the pharmacological effects and mechanisms associated with *A. cochinchinensis* therapeutic effects and to provide a broader vision and new inspiration for research in current and potential applications of *A. cochinchinensis*.

## Botany

**FIGURE 1:**
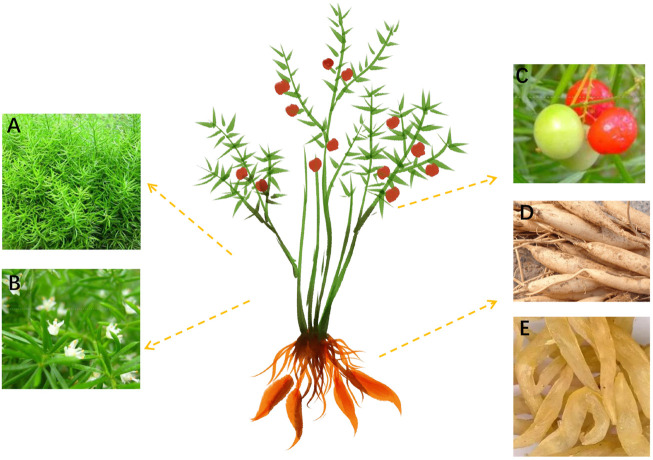
*A. cochinchinensis* plant morphology. **(A)** The above-ground portion, **(B)** Flower, **(C)** Fruits, **(D)** The underground part, **(E)** Medicinal part.

*A. cochinchinensis* is a climbing perennial plant, which has the structural characteristics of pale green stalks, sickle-shaped leaves, pale green axillary flowers, red fruits, and the branches angular or narrowly winged. It usually grows on slopes, roadsides, underwoods, valleys, or wastelands, below 1750 m *A. cochinchinensis* is usually harvested in autumn and winter, cleaned silt, removed fibrous root, retained tuberous root, boiled in boiling water for 15 min, then peeled and cored, further dried to obtain the medicinal part of *A. cochinchinensis*. According to the online records of China’s flora (http://www.cn-flora.ac.cn/index.html), the medicinal part of *A. cochinchinensis* is fusiform, with a swelling in the middle or near the end, which is 3–5 cm long and 1–2 cm thick. *A. cochinchinensis’s stem is* smooth, often curved or twisted, up to 1–2 m long. *A. cochinchinensis’s* leafy branches are usually clustered every 3, which are flat or slightly acute triangular due to the keel shape of the midvein, slightly falcate, 0.5–8 cm long, and 1–2 mm wide. Its inflorescence usually has two axillary flowers with alternate petals. The pedicel is 2–6 mm long. The joint is generally located in the middle, the perianth is 2.5–3 mm long, and the female flowers are similar in size to the male flowers. The flowering and fruiting period is generally from May to October. When the fruit matures, it becomes red, with a diameter of 6–7 mm, with only one seed per fruit, as shown in Figure 1.

## Traditional uses

*A. cochinchinensis* has a long history of ethnopharmacological use and is characterized by bitter in taste and cold in nature. Since ancient times, researchers continuously explore and exploited TCM practices (Zhang X. et al., 2021; Wang et al., 2021). Dating back more than 1700 years of history, *A. cochinchinensis* was first documented in Shennong’s Classic of Materia Medica (Dong Han Dynasty, 25–220 A.D.), which is the earliest classic on TCM. Later, it was listed in many other well-known works on Chinese herb, including “Ming Yi Bie Lu” (Wei and Jin Dynasty, 220–420 A.D.), “Yao Xing Lun” (Tang Dynasty, 618–907 A.D.). In the folk culture, it is often used as a treatment cough, constipation, fever, pneumonia, stomachache, tracheitis, rhinitis, cataract, acne, urticaria and other diseases. In different countries, *A. cochinchinensis* has different therapeutic effects. It can be combined with other herb medicines to achieve a greater therapeutic effect. In Korea, extracts of formulations composed of *A. cochinchinensis* and other herbs were shown to have the effect of treating thrombosis (Chang et al., 2005; Lee et al., 2019). In China, the classic prescription composed of *A. cochinchinensis* (Qisheng pill) contains 114 chemical compounds were identified, including diosgenin, Methyl protodioscin, and ferroic acid, total saponin etc., which can inhibit the occurrence of inflammation, regulate intestinal dysfunction and improve the effect of Alzheimer disease (Xiong et al., 2022). At the same time, the herb formula water decoction composed of *A. cochinchinensis* can treatment of intestinal diseases, especially alleviate allergic airway inflammation and treat asthma (Luo et al., 2020). This also reflects the different therapeutic effects of *A. cochinchinensis* in traditional use and the broad application prospects in the future. Therefore, its clinical efficacy and function still need to be further explored.

## Phytochemistry

**FIGURE 2:**
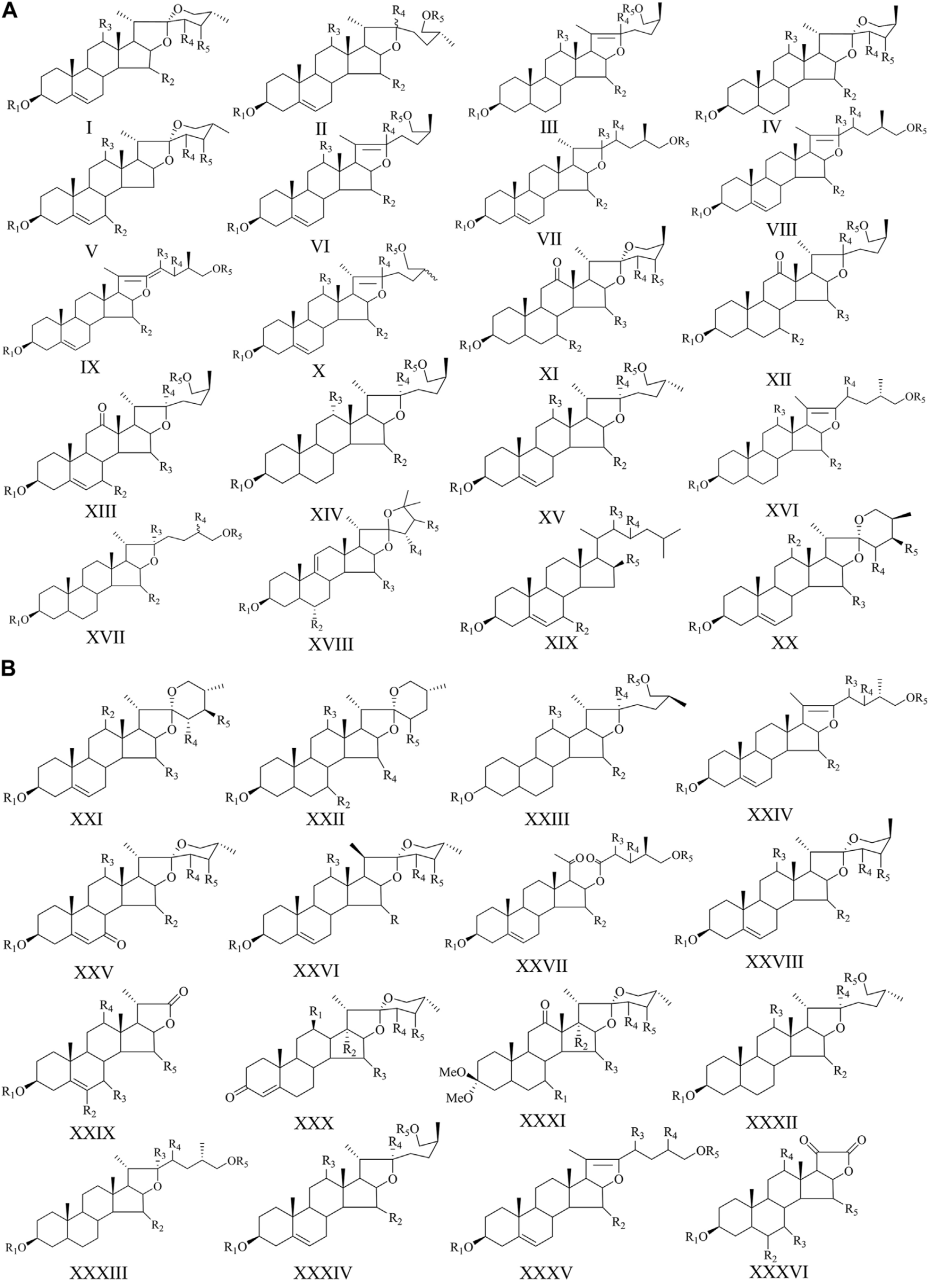
The structures of steroidal saponins (1–71) in *A. cochinchinensis*.

**FIGURE 3:**
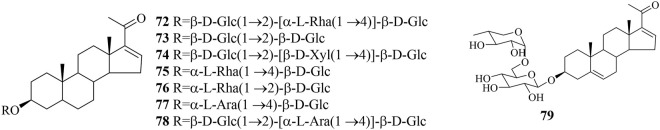
The structures of C_21_-steroidals in *A. cochinchinensis*.

**FIGURE 4:**
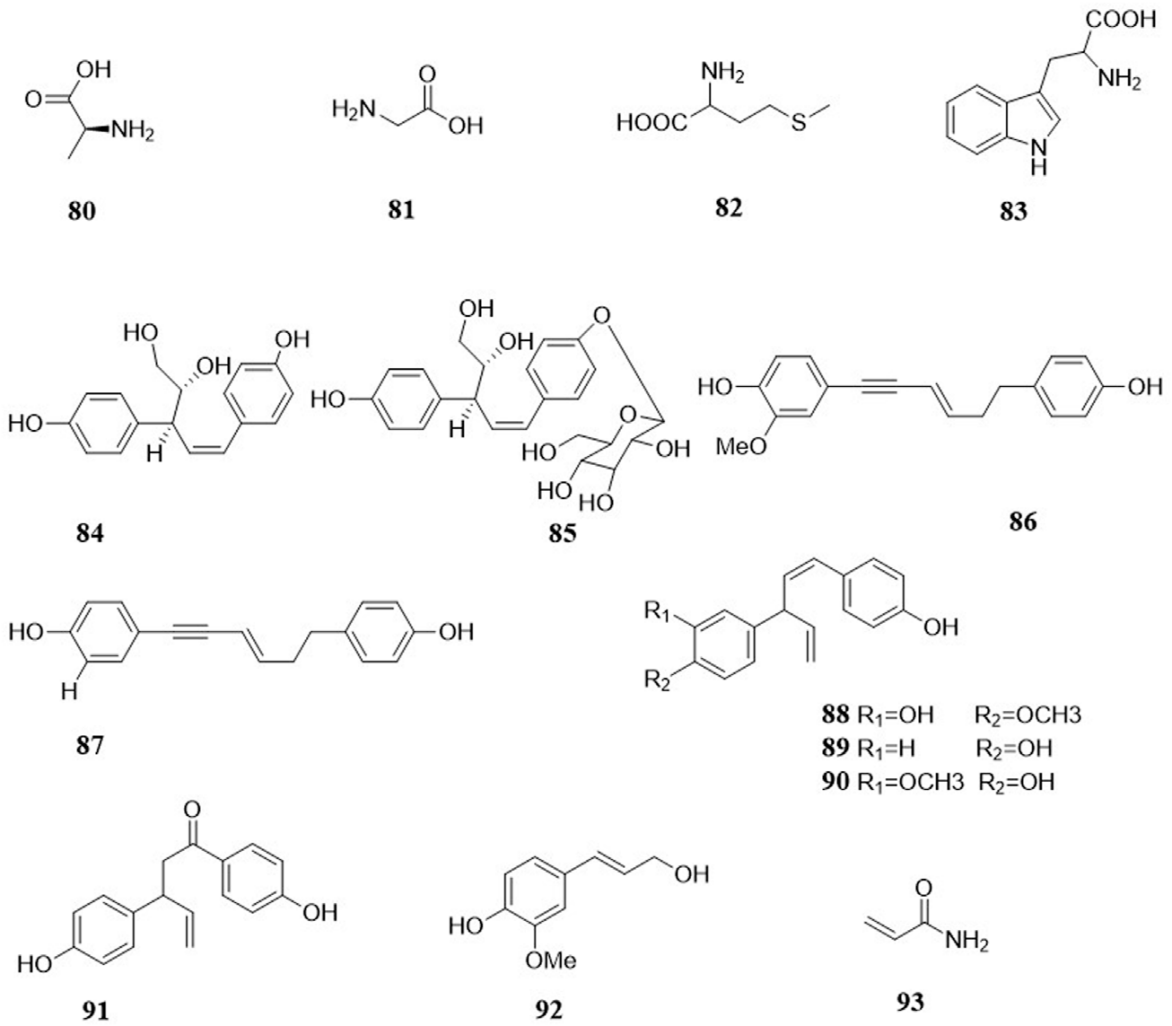
The structures of amino acids, lignans, and other compounds in *A. cochinchinensis.*

In the past few decades, *A. cochinchinensis* have been investigated from a phytochemical perspective. The literature indicates the presence of multiple chemical compounds, predominantly steroidal saponin, C_21_-steroids, amino acids, lignan, and polysaccharides. To date, more than 90 compounds have been isolated and identified from *A. cochinchinensis*. These compounds are summarized in Tables 1 and Table 2, and their structures are shown in Figure 2, and Figure 3, and Figure 4.

**TABLE 1:** Chemical compounds isolated from *A. cochinchinensis.*

Number	Chemical composition	Extraction solvent	Molecular formula	Molecular weight	Reference
Steroidal Saponin					
1	Dioscin	MeOH	C_45_H_72_O_16_	869.0436	Lee et al. (2015)
2	Prosapogenin B	70% EtOH	C_39_H_62_O_12_	722.9024	Liu et al. (2021)
3	(23R, 24R, 25S)-spirost-5-ene-3β,23,24-triol-3-O-α-L-rhamnopyranosyl-(1→2)-[α-L-rhamnopyranosyl-(1→4)]-β-D-glucopyranoside	70% EtOH	C_45_H_72_O_18_	901.0424	Liu et al. (2021)
4	(24S,25S)-spirost-5-ene-3β,24-diol-3-O-α-L-rhamnopyranosyl-(1→2)-[α-L-rhamnopyranosyl-(1→4)]-β-D-glucopyranoside	70% EtOH	C_45_H_72_O_17_	885.0430	Liu et al. (2021)
5	Methylprotodioscin	MeOH	C_52_H_86_O_22_	1063.2260	Liang et al. (1988)
6	(25S)-26-O-β-D-glucopyranosyl-5β-furost-20(22)-en-3β,15β,26-triol-3-O-[α-L-rhamnopyranosyl-(1-4)]-β-D-glucopyranoside	75% EtOH	C_45_H_74_O_17_	887.0589	Shen et al. (2011)
7	Aspacochioside C	75% EtOH; Water	C_45_H_75_O_17_	888.0705	Shen et al., 2011
					Kim et al. (2021)
8	3-O-[α-L-rhamnopyranosyl(1→4)-β-D-glucopyranosyl]- (25S) −5β-spirostan-3β-ol	70% MeOH	C_39_H_64_O_12_	724.9183	Zhu et al. (2021)
9	Asparacoside	MeOH	C_49_H_80_O_21_	1005.1469	Zhang et al. (2004)
10	Nicotianoside B	70% MeOH	C_39_H_64_O_12_	724.9183	Zhu et al. (2021)
11	Immunoside	70% MeOH	-	-	Zhu et al. (2021)
12	Shatavarin IV	70% MeOH	-	-	Zhu et al. (2021)
13	(25S)-5β-spirostan-3β-ol-3-O-α-L-rhamnopyranoside	70% MeOH	C_33_H_54_O_7_	562.7777	Zhu et al. (2014)
14	(25S)-5β-spirostan-3β-ol-3-O-β-D-glucopyranoside	70% MeOH	C_33_H_54_O_8_	578.7771	Zhu et al. (2014)
15	(23S,25R)-23-hydroxyspirost-5-en-3β-yl-O-α-L-rhamnopyranosyl-(1→4)-β-D-glucopyranoside	70% EtOH	C_39_H_62_O_13_	738.9018	Liu et al. (2021)
16	Dioseptemloside F	70% EtOH	C_39_H_62_O_13_	738.9018	Liu et al. (2021)
17	Pseudoprotoneodioscin	75%EtOH; Water	C_51_H_82_O_21_	1031.1842	Shen et al., 2011
18	26-O-β-D-glucopyranosyl-(25R)-furost-5-ene-3β,22α,26-triol 3-O-(1−4)-β-D-glucopyranosyl-α-L-rhamnopyranosyl-(1−2)-[α-L-rhamnopyranosyl-(1−4)]-β-D-glucopyranoside	Water	C_57_H_94_O_27_	1211.3401	Zhang et al. (2021b)
19	Protodioscin	Water; 90% EtOH	C_51_H_84_O_22_	1049.1995	Kim et al. (2021)
					Zhang et al. (2021b)
20	15−hydroxypseudoprotodioscin	Water	C_51_H_82_O_22_	1047.1836	Kim et al. (2021)
21	Dioscoreside H	90% EtOH	C_51_H_82_O_22_	1047.1836	Zhang et al. (2021b)
22	Pseudoprotodioscin	Water	C_51_H_82_O_21_	1031.1842	Liang et al. (1988)
23	(25R)-26-O-β-D-glucopyranosyl-3β,20α,26-trihydroxyfurostan-5,22-diene-3-O-α-L-rhamnopyranosyl-(1→2)-[α-L-rhamnopyranosyl-(1→4)]-O-β-D-glucopyranoside	90% EtOH	-	-	Zhang et al. (2021b)
24	3-O-α-L-rhamnopyranosyl(1→4)-[β-D-glucopyranosyl(1→2)]-β-D-glucopyranosyl-26-O-β-D-glucopyranosyl-(25R)-5β-furostane-3β,22α,26-triol	75% EtOH	C_51_H_86_O_23_	1067.2147	Jian et al. (2013)
25	3-O-β-D-xylopyranosyl(1→4)-[β-D-glucopyranosyl(1→2)]-β-D-glucopyranosyl-26-O-β-D-glucopyranosyl-(25S)-5β-furostane-3β,22α,26-triol	75% EtOH	C_50_H_84_O_23_	1053.1882	Jian et al. (2013)
26	3-O-β-D-glucopyranosyl(1→2)-β-D-glucopyranosyl-26-O-β-D-glucopyranosyl-(25S)-5β-furostane-3β,22α,26-triol	75% EtOH	C_45_H_76_O_19_	921.0735	Jian et al. (2013)
27	3-O-α-L-rhamnopyranosyl (1→4)-[β-D-xylopyranosyl(1→2)]-β-D-glucopyranosyl-26-O-β-D-glucopyranosyl-(25S)-5β-furostane-3β,22α,26-triol	60% EtOH	C_50_H_84_O_22_	1037.1888	Pang et al. (2021)
28	(25S)-26-O-β-D-glucopyranosyl-5β-furostan-3β,22α,26-triol-3-O-α-L-rhamnopyranosyl-(1→4)-β-D-glucopyranoside	70% MeOH	C_45_H_76_O_18_	905.0741	Zhu et al. (2014)
29	(25S)-26-O-β-D-glucopyranosyl-5β-furstan-3β, 22α, 26-triol-3-O-β-D-glucopyranoside	70% MeOH	C_39_H_66_O_14_	758.9329	Zhu et al. (2014)
30	(25S)-5β-12-one-spirost-3β-ol-3-O-β-D-glucopyranoside	60% EtOH	C_33_H_52_O_9_	592.7606	Pang et al. (2021)
31	26-O-β-D-glucopyranosyl-(25S)-5β-12-one-furost-3β,26-diol-3-O-α-L-rhamnopyranosyl-(1→2)-[β-D-xylcopyranosyl-(1→4)]-β-D-glucopyranoside	60% EtOH	C_50_H_82_O_23_	1051.1723	Pang et al. (2021)
32	(25S)-26-O-β-D-glucopyranosyl-5β-furostan-3β,22α,26-triol-12-one-3-O-β-D-glucopyranoside	60% EtOH	C_39_H_64_O_15_	772.9165	Pang et al. (2021)
					Zhu et al. (2014)
33	26-O-β-D-glucopyranosyl-(25S)-Δ^5(6)^-12-one-furost-3β,26-diol-3-O-α-L-rhamnopyranosyl-(1→2)-[β-D-xylcopyranosyl-(1→4)]-β-D-glucopyranoside	60% EtOH	C_50_H_80_O_23_	1049.1564	Pang et al. (2021)
34	26-O-β-D-glucopyranosyl-(25S)-Δ^5(6)^-12-one-furost-3β,26-diol-3-O-α-L-rhamnopyranosyl-(1→2)-[α-L-rhamnopyranosyl-(1→4)]-β-D-glucopyranoside	60% EtOH	C_51_H_82_O_23_	1063.1830	Pang et al. (2021)
35	(25S)-26-O-β-D-glucopyranosyl-22α-methoxy-5β-furostan-3β,26-diol-12-one-3-O-β-D-glucopyranoside	70% MeOH	C_40_H_66_O_15_	786.9430	Zhu et al. (2014)
36	26-O-β-D-glucopyranosyl-(25S)-5β-furost-3β,12α,26-triol-3-O-α-L-rhamnopyranosyl-(1→2)-[α-L-rhamnopyranosyl-(1→4)]-β-D-glucopyranoside	60% EtOH	C_39_H_66_O_15_	774.9323	Pang et al. (2021)
37	Officinalisnin II	60% EtOH	-	-	Pang et al. (2021)
38	(25S)-officinalisnin-I	60% EtOH	C_45_H_76_O_19_	921.0735	Pang et al. (2021)
39	(25S)-26-O-β-D-glucopyranosyl-5β-furostan-3β,22α,26-triol	70% MeOH	C_33_H_56_O_9_	596.7923	Zhu et al. (2014)
40	Pallidifloside I	60% EtOH	C_50_H_82_O_22_	1035.1729	Pang et al. (2021)
41	3-O-[bis-α-L-rhamnopyranosyl-(1→2and1→4)-β-D-glucopyranosyl-25R-furost-5-ene-3β,22α,26-triol]	70% EtOH	C_45_H_74_O_17_	887.0589	Liu et al. (2021)
42	3-O-[{α-L-rhamnopyranosyl-(1→4)}{β-D-glucopyranosyl}]-26-O-[β-D-glucopyranosyl]-(25S)-5β-furost-20(22)-en-3β,26-diol	EtOH	C_45_H_74_O_17_	887.0589	Shi et al. (2004)
43	3-O-β-D-xylopyranosyl(1→4)-[β-D-glucopyranosyl(1→2)]-β-D-glucopyranosyl-26-O-β-D-glucopyranosyl-(25R)-5β-furostane-3β,22α,26-triol	75% EtOH	-	-	Jian et al. (2013)
44	3-O-[{a-L-rhamnopyranosyl-(1→4)} {β-D-glucopyranosyl}]-26-O-[β-D-glucopyranosyl]-(25S)-5β-furostane-3β,22α,26-triol	Water; EtOH	C_45_H_76_O_18_	905.0741	Shi et al. (2004)
45	Chamaedroside E	Water	C_45_H_76_O_19_	921.0735	Kim et al. (2021)
46	Furospirost-5-ene-3β,6α,23α-triol-3-O-α-L-rhamnopyranosyl-(1→4)-β-D-glucopyranoside	70% MeOH	C_40_H_64_O_14_	768.9278	Liu et al. (2021)
47	16β,22,23-trihydroxycholest-5-ene-3β-yl-O-α-L-rhamnopyranosyl-(1→4)-β-D-glucopyranoside	70% MeOH	C_39_H_66_O_13_	742.9335	Liu et al. (2021)
48	(24S,25R)-spirost-5-ene-3β,24-diol-3-O-α-L-rhamnopyranosyl-(1→2)-[α-L-rhamnopyranosyl-(1→4)]-β-D-glucopyranoside	70% MeOH	C_45_H_72_O_17_	884.0430	Liu et al. (2021)
49	(24S,25S)-spirost-5-ene-3β,24-diol-3-O-α-L-rhamnopyranosyl-(1→4)-β-D-glucopyranoside	70% EtOH	C_39_H_62_O_13_	738.9018	Liu et al. (2021)
50	(23S,24R,5S)–23,24-dihydroxyspirost-5-en-3β-yl-O-α-L-rhamnopyranosyl-(1→4)-β-D-glucopyranoside	70% EtOH	C_39_H_62_O_14_	754.9012	Liu et al. (2021)
51	Smilagenin-3-O-α-L-rhamnopyranosyl-(1→4)-β-D-glucopyranoside	70% EtOH	C_39_H_64_O_12_	724.9183	Liu et al. (2021)
52	3-O-{[β-D-glucopyranosyl-(1→2)]-[α-L-rhamnopyranosyl-(1→4)]-β-D-glucopyranosyl} -(25R)-5β-spirostan-3β-ol	70% EtOH	C_45_H_74_O_17_	887.0589	Liu et al. (2021)
53	(25R)-26-[(β-D-glucopyranosyl) oxy]-22α-methoxyfurost-5-en-3β-yl-O-α-L-rhamnopyranosyl-(1→4)-β-D-glucopyranoside	70% EtOH	-	-	Liu et al. (2021)
54	Pseuprotodioscin	70% EtOH	-	-	Liu et al. (2021)
55	Dioscin F	70% EtOH	C_39_H_60_O_13_	736.8859	Liu et al. (2021)
56	Dioscin E	70% EtOH	C_39_H_62_O_12_	722.9024	Liu et al. (2021)
57	3-O-[α-L-rhamnopyranosyl-(1→4)-β-D-glucopyranosyl]-26-O-β-D-glucopyranosyl−20, 22-seco-25R-furoene-20, 22-dione-3β, 26-diol	70% EtOH	C_45_H_74_O_19_	919.0577	Liu et al. (2021)
58	(23S, 24R, 25R)-spirost-5-ene-3β,23,24-triol-3-O-α-L-rhamnopyranosyl-(1→2)-[α-L-rhamnopyranosyl-(1→4)-β-D-glucopyranoside	70% EtOH	C_45_H_72_O_18_	901.0424	Liu et al. (2021)
59	(23R, 25S)-spirost-5-ene-3β, 23-diol-3-O-α-L-rhamnopyranosyl-(1→2)-[α-L-rhamnopyranosyl-(1→4)]-β-D-glucopyranoside	70% EtOH	C_45_H_72_O_17_	885.0430	Liu et al. (2021)
60	Dumoside	70% EtOH	C_40_H_62_O_16_	798.9107	Liu et al. (2021)
61	Asparacosins A	MeOH	C_27_H_40_O_5_	444.6035	Zhang et al. (2004)
62	Asparacosins B	MeOH	C_29_H_46_O_6_	490.6719	Zhang et al. (2004)
63	26-O-β-D-glucopyranosyl-(25R)-5β-furost-3β,26-diol-3-O-α-L-rhamnopyranosyl-(1→2)-[β-D-xylcopyranosyl-(1→4)]-β-D-glucopyranoside	60% EtOH	C_50_H_84_O_22_	1037.1888	Pang et al. (2021)
64	25-*epi*-officinalisnin II	60% EtOH	-	-	Pang et al. (2021)
65	Disporoside C	60% EtOH	C_45_H_76_O_19_	921.0735	Pang et al. (2021)
66	26-O-β-D-glucopyranosyl-(25R)-5β-furost-3β,26-diol-3-O-α-L-rhamnopyranosyl-(1→2)-[β-D-xylcopyranosyl-(1→4)]-[α-L-rhamnopyranosyl-(1→6)]-β-D-glucopyranoside	60% EtOH	C_56_H_94_O_27_	1199.3294	Pang et al. (2021)
67	26-O-β-D-glucopyranosyl-(25R)-5β-furost-3β,26-diol-3-O-α-L-rhamnopyranosyl-(1→2)-[α-L-rhamnopyranosyl-(1→4)]-[α-L-rhamnopyranosyl-(1→6)]-β-D-glucopyranoside	60% EtOH	C_57_H_96_O_27_	1213.3560	Pang et al. (2021)
68	3-O-[{α-L-rhamnopyranosyl-(1→4)} {β-D-glucopyranosyl}]-26-O-[β-D-glucopyranosyl]-22α-methoxy-(25S)-5β-furostane-3β,26-diol	EtOH	C_46_H_79_O_18_	904.1131	Shi et al. (2004)
69	Protoneodioscin	60% EtOH	-	-	Pang et al. (2021)
70	3-O-[a-L-rhamnopyranosyl-(1→4) β-D-glucopyranosyl]-26-O-(β-D-glucopyranosyl) -(25R)-furosta-5,20-diene, -3β,26-diol	Water	-	-	Liang et al., 1988
					Liu et al. (2021)
71	5β-pregn-20-ene-3,16-diol-22-one 3-O-α-L-rhmnopyranosyl-(1→2)-β-D-glucopyranoside	70% MeOH	C_34_H_52_O_12_	652.7695	Zhu et al. (2021)
C^21^-steroide					
72	3-O-β-D-xylopyranosyl(1→4)-[β-D-glucopyranosyl(1→2)]-β-Dglucopyranosyl-5β-pregna-16-ene-33β-ol-20-one	75% EtOH	C_38_H_60_O_16_	772.8734	Jian et al. (2013)
73	3-O-α-L-rhamnopyranosyl (1→4)- [β-D-glucopyranosyl (1→2)]-β-D-glucopyranosyl-5β-pregna-16-ene-3β-ol-20-one	75% EtOH	C_33_H_52_O_12_	640.7588	Jian et al. (2013)
74	3-O-β-D-glucopyranosyl (1→2)-β-D-glucopyranosyl-5β-pregna-16-ene-3β-ol-20-one	75% EtOH	C_33_H_52_O_12_	640.7588	Jian et al. (2013)
75	(3β,5β)-pregn-16(17)-en-3-ol-20-one 3-O-α-L-rhmnopyranosyl-(1→4)-β-D-glucopyranoside	70% MeOH	C_33_H_52_O_11_	624.7594	Zhu et al. (2021)
76	(3β,5β)-pregn-16(17)-en-3-ol-20-one 3-O-α-L-rhmnopyranosyl-(1→2)-β-D-glucopyranoside	70% MeOH	C_33_H_52_O_11_	624.7594	Zhu et al. (2021)
77	(3β,5β)-pregn-16(17)-en-3-ol-20-one 3-O-α-L-arabinopyranosyl-(1→4)-β-D-glucopyranoside	70% MeOH	C_20_H_50_O_11_	466.6040	Zhu et al. (2021)
78	(3β,5β)-pregn-16(17)-en-3-ol-20-one 3-O-α-L-arabipyrannosyl-(1→4)-β-D-glucopyranosyl-(1→2)-β-D-glucopyranosid	70% MeOH	C_38_H_60_O_16_	772.8734	Zhu et al. (2021)
79	3β-[(O-α-L-rhamnopyranosyl-(1→4)-β-D-glucopyranosyl)oxy]pregna-5,-16-dien-20-one	70% EtOH	C_33_H_50_O_11_	622.7435	Liu et al. (2021)
Amino acid					
80	Alanine	Water	C_3_H_7_NO_2_	89.0932	Choi et al., 2019
81	*Glycine*	Water	C_2_H_5_NO_2_	75.0666	Choi et al., 2019
82	Methionine	Water	C_5_H_11_NO_2_S	149.2113	Choi et al., 2019
83	Tryptophan	Water	C_11_H_12_N_2_O_2_	204.2252	Choi et al., 2019
Lignan					
84	Iso-agatharesinol	70% EtOH	C_17_H_18_O_4_	286.3224	Li et al. (2012)
85	Iso-agatharesinoside	70% EtOH	C_23_H_28_O_9_	448.4630	Li et al. (2012)
Others					
86	1-[4-hydroxyphenoxy]-5-[3-methoxy-4-hydroxyphenyl] pent-2-en-3-yne	MeOH	C_18_H_16_O_4_	296.3172	Zhang et al. (2004)
87	Asparenydiol	MeOH	C_17_H_13_O_3_	265.2839	Zhang et al. (2004)
88	3′-hydroxy-4′-methoxy-4′-dehydroxynyasol	MeOH	C_18_H_18_O_3_	282.3337	Zhang et al. (2004)
89	Nyasol	MeOH	C_17_H_16_O_2_	252.3077	Zhang et al. (2004)
90	3″-methoxynyasol	MeOH	C_17_H_16_O_3_	268.3071	Zhang et al. (2004)
91	1,3-bis-di-p-hydroxyphenyl-4-penten-1-one	MeOH	C_17_H_16_O_3_	268.3071	Zhang et al. (2004)
92	Trans-coniferyl alcohol	MeOH	C_10_H_12_O_3_	180.2005	Zhang et al. (2004)
93	Acrylamide	Water	C_3_H_5_NO	71.0779	Shi et al. (2009)

**TABLE 2:** The structures of steroidal saponins in *A. cochinchinensis.*

NO	Structure					
	Mother nucleus	R_1_	R_2_	R_3_	R_4_	R_5_
1	I	α-L-Rha (1→2)-[α-L-Rha (1→4)]-β-D-Glc	H	H	H	H
2	I	α-L-Rha (1→4)-β-D-Glc	H	H	H	H
3	I	α-L-Rha (1→2)-[α-L-Rha (1→4)]-β-D-Glc	H	H	OH	OH
4	I	α-L-Rha (1→2)-[α-L-Rha (1→4)]-β-D-Glc	H	H	H	OH
5	II	α-L-Rha (1→2)- [α-L-Rha (1→4)]-β-D-Glc	H	H	OCH_3_	β-D-Glc
6	III	α-L-Rha (1→4)-β-D-Glc	OH	H	H	β-D-Glc
7	III	α-L-Rha (1→4)-β-D-Glc	H	H	H	β-D-Glc
8	IV	α-L-Rha (1→4)-β-D-Glc	H	H	H	H
9	IV	β-D-Glc(1→2)-[α-L-Ara(1→4)]-[a-L-Ara(1→6)]-β-D-Glc	H	H	H	H
10	IV	α-L-Rha (1→2)-β-D-Glc	H	H	H	H
11	IV	α-L-Rha(1→2)-[α-L-Rha(1→4)]-β-D-Glc	H	H	H	H
12	IV	β-D-Glc(1→2)-[α-L-Rha(1→4)]-β-D-Glc	H	H	H	H
13	IV	α-L-Rha	H	H	H	H
14	IV	β-D-Glc	H	H	H	H
15	V	α-L-Rha (1→4)-β-D-Glc	H	H	OH	H
16	V	α-L-Rha (1→4)-β-D-Glc	α-H,β-OH	H	H	H
17	VI	α-L-Rha(1→2)-[β-D-Glc(1→4)]-β-D-Glc	H	H	H	β-D-Glc
18	VII	α-L-Rha(1→2)-[β-D-Glc(1→4)-α-L-Rha(1→4)]-β-D-Glc	H	H	OH	β-D-Glc
19	VII	α-L-Rha(1→2)-[α-L-Rha(1→4)]-β-D-Glc	H	H	OH	β-D-Glc
20	VIII	α-L-Rha(1→2)-[α-L-Rha(1→4)]-β-D-Glc	OH	H	H	β-D-Glc
21	VIII	α-L-Rha(1→2)-[α-L-Rha(1→4)]-β-D-Glc	H	H	OH	β-D-Glc
22	VIII	α-L-Rha(1→2)-[α-L-Rha(1→4)]-β-D-Glc	H	H	H	β-D-Glc
23	IX	α-L-Rha(1→2)-[α-L-Rha(1→4)]-β-D-Glc	H	H	H	β-D-Glc
24	X	β-D-Glc(1→2)-β-D-Glc	H	H	OCH_3_	β-D-Glc
25	X	α-L-Rha (1→4)-β-D-Glc	H	O	OH	β-D-Glc
26	X	β-D-Glc(1→2)-[α-L-Rha(1→4)]-β-D-Glc	H	H	OH	β-D-Glc
27	X	β-D-Xyl(1→2)-[α-L-Rha(1→4)]-β-D-Glc	H	H	OH	β-D-Glc
28	X	α-L-Rha (1→4)-β-D-Glc	H	H	OH	β-D-Glc
29	X	β-D-Glc	H	H	OH	β-D-Glc
30	XI	β-D-Glc	H	H	H	H
31	XII	α-L-Rha(1→2)-[β-D-Xyl(1→4)]-β-D-Glc	H	H	OH	β-D-Glc
32	XII	β-D-Glc	H	H	H	β-D-Glc
33	XIII	α-L-Rha(1→2)-[β-D-Xyl(1→4)]-β-D-Glc	H	H	OH	β-D-Glc
34	XIII	α-L-Rha(1→2)-[α-L-Rha(1→4)]-β-D-Glc	H	H	OH	β-D-Glc
35	XIII	β-D-Glc	H	H	OCH_3_	β-D-Glc
36	XIV	β-D-Glc	H	OH	OH	β-D-Glc
37	XIV	β-D-Glc(1→2)-[β-D-Xyl(1→4)]-β-D-Glc	H	H	OH	β-D-Glc
38	XIV	β-D-Glc(1→2)-β-D-Glc	H	H	OH	β-D-Glc
39	XIV	H	H	H	OH	β-D-Glc
40	XV	α-L-Rha(1→2)-[β-D-Xyl(1→4)]-β-D-Glc	H	H	OH	β-D-Glc
41	XV	α-L-Rha(1→2)-[α-L-Rha(1→4)]-β-D-Glc	H	H	OH	β-D-Glc
42	XVI	α-L-Rha (1→4)-β-D-Glc	H	H	H	β-D-Glc
43	XVII	β-D-Glc(1→2)-[β-D-Xyl(1→4)]-β-D-Glc	H	OH	CH_3_	β-D-Glc
44	XVII	α-L-Rha(1→4)-β-D-Glc	H	OH	β-methyl	β-D-Glc
45	XVII	β-D-Glc(1→4)-β-D-Glc	H	OH	α-methyl	β-D-Glc
46	XVIII	α-L-Rha (1→4)-β-D-Glc	OH	H	OH	H
47	XIX	α-L-Rha (1→4)-β-D-Glc	H	OH	OH	OH
48	XX	α-L-Rha(1→2)-[α-L-Rha(1→4)]-β-D-Glc	H	H	H	OH
49	XXI	α-L-Rha (1→4)-β-D-Glc	H	H	H	OH
50	XXI	α-L-Rha (1→4)-β-D-Glc	H	H	OH	OH
51	XXII	α-L-Rha (1→4)-β-D-Glc	H	H	H	H
52	XXII	β-D-Glc(1→2)-[α-L-Rha(1→4)]-β-D-Glc	H	H	H	H
53	XXIII	β-D-Glc(1→4)-β-D-Glc	H	H	OCH_3_	β-D-Glc
54	XXIV	α-L-Rha(1→2)-[α-L-Rha(1→4)]-β-D-Glc	H	H	H	β-D-Glc
55	XXV	α-L-Rha (1→4)-β-D-Glc	H	H	H	H
56	XXVI	α-L-Rha (1→4)-β-D-Glc	H	H	H	H
57	XXVII	α-L-Rha (1→4)-β-D-Glc	H	H	H	β-D-Glc
58	XXVIII	α-L-Rha(1→2)-[α-L-Rha(1→4)]-β-D-Glc	H	H	OH	OH
59	XXVIII	α-L-Rha(1→2)-[α-L-Rha(1→4)]-β-D-Glc	H	H	OH	H
60	XXIX	α-L-Rha(1→2)-[α-L-Rha(1→4)]-β-D-Glc	H	H	H	H
61	XXX	OH	OH	H	H	H
62	XXXI	H	OH	H	H	H
63	XXXII	α-L-Rha(1→2)-[β-D-Xyl(1→4)]-β-D-Glc	H	H	OH	β-D-Glc
64	XXXII	β-D-Glc(1→2)-[β-D-Xyl(1→4)]-β-D-Glc	H	H	OH	β-D-Glc
65	XXXII	β-D-Glc(1→2)-β-D-Glc	H	H	OH	β-D-Glc
66	XXXII	β-D-Glc(1→2)-[β-D-Xyl(1→4)]-[α-L-Rha(1→6)]-β-D-Glc	H	H	OH	β-D-Glc
67	XXXII	β-D-Glc(1→2)-[α-L-Rha(1→4)]-[α-L-Rha(1→6)]-β-D-Glc	H	H	OH	β-D-Glc
68	XXXIII	α-L-Rha (1→4)-β-D-Glc	H	OCH_3_	H	β-D-Glc
69	XXXIV	α-L-Rha(1→2)-[α-L-Rha(1→4)]-β-D-Glc	H	H	OH	β-D-Glc
70	XXXV	α-L-Rha (1→4)-β-D-Glc	H	H	CH_3_	β-D-Glc
71	XXXVI	α-L-Rha (1→2)-β-D-Glc	H	H	H	H

### Steroidal saponins

Steroidal saponins are the major chemical components in *A. cochinchinensis* (Lee et al., 2015). Thus far, 71 steroidal saponins (1–71) have been isolated from *A. cochinchinensis* in Table 2. Steroid saponins are mainly composed of steroidal saponins and sugar condensation. They are classified into spirostanol saponins, isosprirostanol saponins, pseudospirostanol saponins and furostanol saponins based on the aglycone component differences. Aglycones are composed of six rings, of which the rutile rings are usually connected in a spiroketal form. The sugar moieties in the ordinary steroidal saponins are attached to the hydroxyl groups at C_3_. In a word, the structural diversity of different compounds is more reflected in the kind, length of each monosaccharide, the type of glycoside bond at the C_3_ position, and the position of the substituent.

### C_21_-steroides

C_21_-steroides are steroid derivatives with 21 carbon atoms and are one of the key compounds in *A. cochinchinensis*. C_21_ steroids are mostly hydroxyl derivatives with pregnane or its isomers as the basic skeleton. According to the skeleton type, they can be divided into four types, of which 72–79 (Jian et al., 2013; Liu et al., 2021; Zhu et al., 2021) are typical C_21_-steroides in Figure 3. In addition, there are many hydroxyl and carbonyl groups on the C_21_-steroid mother nucleus, and most of the carbonyl groups are at C_20_.

### Amino acids

Four kinds of amino acids were isolated from *A. cochinchinensis* 80–83 (Choi et al., 2019), and their structures are shown in Figure 4. Amino acids are compounds containing both amino and carboxyl groups. In terms of their structure, amino acids are derivatives of carboxylic acid molecules in which amino groups replace the hydrogen in the alkyl group. According to the relative number of amino and carboxyl groups in amino acid molecules, amino acids can be divided into neutral, acidic and basic.

### Lignans

Lignans are a kind of natural compounds synthesized by the polymerization of two-molecular phenylpropanoid derivatives, most of which are free, and a few are glycosides bound to a sugar. At present, a small concentration of lignans 84–85 (Li et al., 2012) was identified from *A. cochinchinensis.* Compared with other compounds, lignans have less structure. Therefore, future efforts should be made to isolate and characterize lignans in *A. cochinchinensis*.

### Polysaccharides

In recent years, plant polysaccharides have attracted high research interest due to their unique biological activity and natural origin, with great potential to protect human health. Many natural products are is rich in polysaccharide resources, especially medicinal plant polysaccharides, with long application history and broad development prospects. *A. cochinchinensis* polysaccharides are mainly comprised of Man, Rha, Glc, Gal, Ara, Xyl, Fru, GlcUA, and GalUA, as shown in Table 3.

**TABLE 3:** Composition and analysis of polysaccharides in *A. cochinchinensis.*

No	Name	Extraction	Analytical method	Analytical condition	Monosaccharide composition	Molecular weight (Da)	Main structure	Reference
1	ACNP	distilled water refluxed (6h)	HPLC	Sugar-pack ™column (6.5 mm × 300 mm, 10 μm) and ELSD (evaporative light scattering detector); distilled water; 0.4 ml/min; column temperature 30°C	Fru, Glc	2690	2,1-*β-D-Fruf* residues, ending with a (1→2) bonded *α-D-Glcp*	Sun et al. (2020)
			GC-MS		93.3: 6.7(area %)			
2	Radix Asparagi polysaccharide	deionized water refluxed (4.5h)	CZE	40 mM sodium tetraborate buffer (pH 10.1); hydrodynamic injection (10 cm × 4 s); 14 kV	Xyl, Ara, Glc, Rha, Man, Gal, GlcUA, GalUA	-	-	Chen et al. (2015)

### Other compounds

In addition to the five major phytochemical compound classes mentioned above, other bioactive constituents have also been isolated from *A. cochinchinensis* (Zhang et al., 2004; Shi et al., 2009). These include 1-[4-hydroxyphenoxy]-5-[3-methoxy-4-hydroxyphenyl] pent-2-en-3-yne (86), asparenydiol (87),3′-hydroxy-4′-methoxy-4′dehydroxynyasol (88), Nyasol(89), 3″-methoxynyasol(90), 1,3-bis-di-p-hydroxyphenyl-4-penten-1-one(91), trans-coniferyl alcohol(92), Acrylamide(93). The above findings illustrate the wide chemical composition of *A. cochinchinensis*, which is of immense future research value.

## Pharmacological activities

**FIGURE 5:**
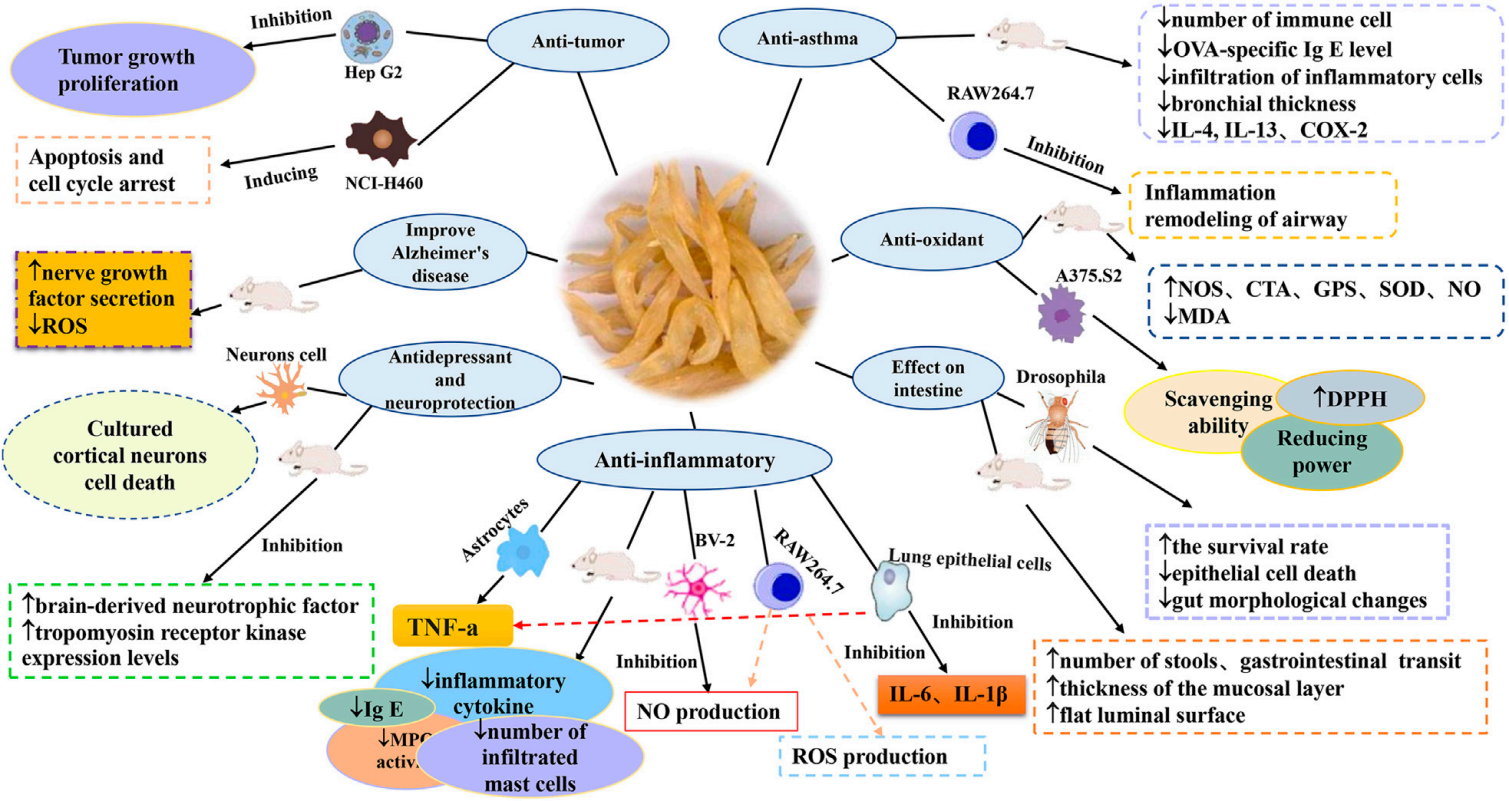
The pharmacological activities of *A. cochinchinensis.*

*A. cochinchinensis* exerts various pharmacological activities, including anti-asthma, anti-inflammation, anti-oxidant, anti-tumor, anti-depressant, neuroprotective, improve Alzheimer’s disease and gut diseases. To illustrate the nature of the active compounds of *A. cochinchinensis*, the pharmacological effects and potential mechanisms of this plant on the basis of different types of extracts and compounds were summarized in Table 4. A simplified diagram of its pharmacological effects is presented in Figure 5.

**TABLE 4:** Summary of pharmacological activities of *A. cochinchinensis* extracts/compounds.

Pharmacological activities	Extracts/Compounds	Models	Results/Mechanisms	Dosages	References
Anti-asthma	Water extract (2.5 h)	Mice (OVA-induced)	↓Number of immune cells, ↓OVA-specific Ig E level, ↓thickness of respiratory epithelium and mucus score	500 mg⋅kg^−1^	Choi et al. (2018b)
	Water extract	Mice (OVA-induced)	Prevent inflammation and remodeling of airway	250 and 500 mg⋅kg^−1^	Choi et al. (2019)
	Water extract (2.5 h)	Mice (OVA-induced)	↓Infiltration of inflammatory cells and bronchial thickness; ↓number of macrophages and eosinophils, ↓concentration of OVA-specific Ig E, and expression of Th2 cytokines	250 and 500 mg⋅kg^−1^	Choi et al. (2018a)
	Total saponin	Mice (OVA-induced); RAW264.7 cells (LPS-activated)	↓Number of immune cells, ↓infiltration of inflammatory cells, ↓bronchial thickness, ↓IL-4, IL-13 and COX-2	250 and 500 mg⋅kg^−1^; 200 µg⋅mL^−1^	Sung et al. (2017)
Anti-inflammatory	Distilled water extract (70°C for 5 h)	Astrocytes (stimulated with SP and LPS)	Inhibit TNF-alpha secretion by inhibiting IL-1 secretion	10^1^–10^3^ µg⋅mL^−1^	Kim et al. (1998)
	70% EtOH extract (three times, with 2 h reflux)	Mice (TPA-induced)	↓Skin thickness and tissue weight, ↓inflammatory cytokine production, ↓neutrophil-mediated MPO activity	200 mg⋅kg^−1^	Lee et al. (2009)
	Ethyl acetate extract (three times, with 2 h reflux)	Mice (IL-4/Luc/CNS-1 Tg)	↓Immunoglobulin E concentration, ↓epidermis thickness, ↓number of infiltrated mast cells	200 and 400 mg⋅kg^−1^	Sung et al. (2016)
	Ethyl acetate extract (50°C for 24 h)	RAW264.7 cells (LPS-activated)	Inhibition of NO production, COX-2 expression, ROS production, differential regulation of inflammatory cytokines cell cycle	100 and 200 µg⋅mL^−1^	Lee et al. (2017b)
	Methyl Protodioscin	Lung epithelial cells; Mice (airway inflammation)	Inhibited the production of proinflammatory cytokines IL-6, TNF-α, IL-1β in lung tissue	10–100 μM	Lee et al. (2015)
	Butanol extract (three times)	RAW264.7 macrophage cells (LPS-stimulated)	Inhibition of proinflammatory cytokine expression	100 and 200 µg⋅mL^−1^	Lee et al. (2017b)
	75% EtOH (three times, 3 h at 70°C)	BV-2 microglial cells (LPS-induced)	Inhibition of NO production	1.0 μg⋅mL^−1^	Jian et al. (2013)
Anti-oxidant	Water extract (three times)	Mice (D-galactose-induced aging)	↑NOS, CAT, SOD activities, ↑NO content, ↓ MDA content	0.7 g⋅mL^−1^	Lei et al. (2017)
	Water extract (three times)	Mice (D-galactose-induced aging)	↑NOS, CAT, SOD activities and the NO content; ↑expressions of NOS, ↑SOD and GPX	0.7 g⋅mL^−1^	Lei et al. (2016)
	25% ethyl acetate extract (three times, 40°C for 2 h)	CCD-966SK cell; A375.S2 cell	↑Scavenging ability, reducing power,↑anti-tyrosinase activity of DPPH	100–1000 mg⋅L^−1^	Wang et al. (2019)
	Water extract (1 h three times)	Mice (D-Galactose)	↑Spleen index and the SOD activity; ↓MDA content	2.66 g⋅kg^−1^	Xiong et al. (2011)
Anti-tumor	90% EtOH extract (80°C for 3 h)	Hep G2 cells, Hep 3B cell, LO 2 cell; mice (Tumor-Bearing)	Inhibit tumor growth and proliferation	200 mg⋅kg^−1^	Zhang et al. (2021b)
	70% EtOH extract (refluxing three times, 2 hours each time)	NCI-H460 cell	Inducing apoptosis and cell cycle arrest; inhibition of lung cancer cell proliferation	10, 50 and 100 μM	Liu et al. (2021)
	Water extract (decoction 3 h)	Hep G2 cells	Inhibited the TNF-alpha-induced apoptosis of Hep G2 cells	1–100 mg⋅mL^−1^	Koo et al. (2000)
Antidepressant and neuroprotection	Water extract (100°C for 2 h)	Mice (Ovariectomized)	↑Brain-derived neurotrophic factor	1000 and 2000 mg⋅kg^−1^	Kim et al. (2020)
			↑Tropomyosin receptor kinase expression levels		
	MeOH extract (5 days)	Cortical neurons cell	Inhibited H2O2-induced cell death in cultured cortical neurons	0.01, 0.50 and 1.00 μM; 100 and 200 mg⋅kg^−1^	Jalsrai et al. (2016)
		Mice			
Treat intestinal related diseases	Water extract (3 h, repeated twice)	*Drosophila*	↑The survival rate; ↓epithelial cell death; attenuated metal ion-induced gut morphological changes	10% w⋅v^−1^	Zhang et al. (2016)
	Saponin (24 h at 50°C)	Mice (loperamide-induced constipation)	↑Number of stools and gastrointestinal transit, ↑thickness of the mucosal layer, ↑flat luminal surface, ↑number of paneth cells,↑lipid droplets	1000 mg⋅kg^−1^	Kim et al. (2019)
Improve Alzheimer’s disease	Water extract (121°C for 45 min)	Mice	↑Nerve growth factor secretion; ↓intracellular ROS	100 mg⋅kg^−1^	Lee et al. (2018)

### Anti-asthma

Asthma is a common chronic and stubborn respiratory disease, clinically presenting with cough, chest tightness, wheezing, and shortness of breath (Papi et al., 2018). Non-timely treatment will lead to a series of secondary diseases, such as chronic obstructive pulmonary disease and heart failure, which can become life-threatening (Schoettler and Strek, 2020; Miller et al., 2021). At the same time, it also added a serious financial burden to the family (López-Tiro et al., 2022). Therefore, researchers found that the butanol extract of *A. cochinchinensis* roots, when fermented with Weissella cibaria (BAfW), was found to inhibit the development of asthma development through various potential mechanisms. Choi et al., 2018 alterations in key parameters were measured in ovalbumin (OVA)-challenged Balb/c mice treated with different BAfW dose regimens at three different time points. The results show that when the dosage of *A. cochinchinensis* fermentation extract was 500 mg, the number of immune cells, OVA-specific immunoglobulin E (Ig E) level, thickness of respiratory enzyme and mucus score decreased significantly in mice, and these parameters could be maintained for 48 h (Choi et al., 2018b). At the same time, researchers explored biomarkers for asthma in OVA-induced asthma mice. The extract of *A. cochinchinensis* was administered to the model mice at a low concentration of 250 mg/kg and a high concentration of 500 mg/kg, respectively. The changes in their metabolites were observed after administration. The results showed that the immune cells, Ig E serum concentration, the respiratory epithelium’s thickness, and inflammatory cell infiltration in the airway in mice treated with *A. cochinchinensis* extract recovered significantly. Notably, when assessing the endogenous metabolites, only alanine, glycine, methionine, and tryptophan were significantly recovered after *A. cochinchinensis* extract treatment, compared with the control group. Therefore, these four metabolites can be used as biomarkers to predict the anti-asthmatic effects (Choi et al., 2019). Moreover, the *A. cochinchinensis* fermentation extract was shown for the first time to accelerate the recovery from chronic asthma. It prevented airway inflammation and remodeling by restoring the cholinergic regulation of structural cells and inflammatory cells in chronic asthma models. Thus, it can further potentiate the effects of asthma treatments (Choi et al., 2018a). Furthermore, *in vitro* and *in vivo* experiments have been conducted to explore the effects of total saponins in *A. cochinchinensis* extract on asthma. Lipopolysaccharide (LPS) -activated RAW264.7 cells and OVA-induced mice asthma were treated with saponins-rich *A. cochinchinensis* extract, respectively. The result showed that the concentration of nitric oxide (NO) and mRNA levels of and cyclooxygenase-2 (COX-2) and inducible nitric oxide synthase (iNOS) were significantly decreased in the SEAC/LPS-treated RAW264.7 cells compared with the vehicle/LPS-treated RAW264.7 cells. At the same time, the number of immune cells, infiltration of inflammatory cells and bronchial thickness decreased, meanwhile the levels of interleukin 4 (IL-4), interleukin 13 (IL-13) decreased significantly under the treatment of *A. cochinchinensis* extract (Sung et al., 2017). In general, *A. cochinchinensis* extracts can inhibit airway inflammation and remodeling, providing an important natural medicine option for the treatment of asthma.

### Anti-inflammatory

Inflammation commonly occurs due to the modern lifestyle, and its complications can detrimentally affect people’s health (Yeung et al., 2018; McInnes and Gravallese, 2021). Numerous studies have proved that *A. cochinchinensis* has anti-inflammatory effect. Previous research by Kim et al., 1998 showed that *A. cochinchinensis* could inhibit tumor necrosis factor-α (TNF-α) secretion by inhibiting interleukin 1 (IL-1) secretion and that *A. cochinchinensis* extracts had anti-inflammatory activity in the central nervous system (Kim et al., 1998). Another study showed that the ethanol extract of *A. cochinchinensis* inhibited acute and chronic inflammation. When the extract was administered at a 200 mg/kg dose, the symptoms of 12-o-tetradecanoyl-phorbol-13-acetate (TPA)-induced mice ear were significantly alleviated. In addition, the skin thickness and tissue weight, inflammatory cytokine production, neutrophil-mediated myeloperoxidase (MPO) activity and histopathological parameters were significantly decreased (Lee et al., 2009). Furthermore, researchers have found that the ethyl acetate extract of *A. cochinchinensis* was shown to inhibits skin inflammation. In this study, phthalic anhydride (PA) -induced skin inflammation mice were used to identify the effects of *A. cochinchinensis* ethyl acetate extract on inflammation. The results suggest that ethyl acetate extract of *A. cochinchinensis* significantly reduced the concentration of Ig E, the surface thickness and number of infiltrating mast cells, and ethyl acetate extract played a key role in the treatment process (Sung et al., 2016). Using *in vitro* cell experiments, the researchers showed that the *A. cochinchinensis* ethyl acetate extract could inhibit the LPS stimulated RAW264.7 cell NO production, COX-2 expression, reactive oxygen species (ROS) production, and the inflammatory cytokine cell cycle (Lee et al., 2017). Thus, the above research findings provide strong evidence that *A. cochinchinensis* extracts may have important medicinal properties for treating specific skin inflammatory diseases. Surprisingly, after fermentation with BAfW, compounds such as protodioscin were significantly enhanced. In addition, a significant suppression was observed in the expression of key members of the iNOS-mediated COX-2 induction pathway and the phosphorylation of mitogen-activated protein kinases. These observations point to the ability to inhibit inflammatory reaction occurrence (Lee et al., 2015). Furthermore, studies have shown that the compound methyl protodioscin in *A. cochinchinensis* can inhibit the production of pro-inflammatory factors such as interleukin 16 (IL-16), interleukin 8 (IL-8) and TNF-α in lung tissue, suggesting that the compound has therapeutic value for airway inflammatory diseases (Lee et al., 2017a). Additionally, through *in vitro* cell experiments, the researchers took LPS-induced microglia cell as the study model. They were found that the ethanol extract of *A. cochinchinensis* at 1.0 μg mL^−1^ could significantly inhibit the production of NO in microglia cell induced by LPS, so as to play an anti-inflammatory role (Jian et al., 2013). All in all, all these studies have emphasized the potential of *A. cochinchinensis* extract to inhibit inflammatory reactions.

### Anti-oxidant

Anti-oxidants have always played a vital role in people’s health (Milisav et al., 2018; Martemucci et al., 2022). Studies have recently confirmed the anti-oxidant effect of *A. cochinchinensis* extract. *A. cochinchinensis* is shown to significantly increase the activities of anti-oxidant enzymes such as superoxide dismutase (SOD), catalase (CAT), nitric oxide synthase (NOS), NO, and glutathione peroxidase (GPX). Liver and kidney hematoxylin and eosin stain sections revealed that D-galactose could cause serious injury, and *A. cochinchinensis* treatment improved immunity and substantially protected the liver and kidney from oxidative damage in aging mice (Lei et al., 2016). In a similar experiment, compared with the Vitamin C (Vc) positive control group, 0.7 mg⋅mL^−1^ aqueous root extract of *A. cochinchinensis* had similar 1,1-Diphenyl-2-picrylhydrazyl (DPPH) and 3-ethylbenzothiazoline-6-sulfonic (ABTS^+^) scavenging activities, but significantly increased superoxide anion (*p* < 0.05) and OH scavenging activities (*p* < 0.01), which suggested strong radical scavenging ability of the aqueous root extract *in vitro* (Lei et al., 2017). At the same time, the researchers took D-galactose -induced mice as the research object and administered intraperitoneal injection (0.2 ml/20g) to mice for 15 days to make them senile, and further explored the effect of *A. cochinchinensis* extract on aging mice. The study found that through the detection of mice spleen and plasma, *A. cochinchinensis* extract could increase the spleen index and the SOD activity, reduces malondialdehyde **(**MDA) content, inhibits oxidation and slows down aging (Xiong et al., 2011). Additionally, 2,2-diphenyl-1-picropylhydrazine (DPPH) plays an indispensable role in antioxidant process. In a recent study, the fermented *A. cochinchinensis* root extract’s effects on melanogenic factor levels in human epidermal melanocytes (HEMs) and its anti-tyrosinase activity were analyzed and compared with the unfermented extract. The results showed that the scavenging ability, reducing power, and anti-tyrosinase activity of DPPH in the fermented extract were significantly increased (Wang et al., 2019). Therefore, *A. cochinchinensis* can be used as a natural anti-oxidant, with broad development and application prospects in the future.

### Anti-tumor

The prevention and treatment of malignant tumors and cancer is a major challenge faced in our modern societies (Liu and Dong, 2021; DiMaio et al., 2022; Mao et al., 2022). With the development of molecular biology and pharmacology, *A. cochinchinensis* has attracted increasing attention from domestic and foreign medical scholars working in the cancer field. Through *in vitro* and *in vivo* experiments, *A. cochinchinensis* extracts were mainly internalized into tumor cells through phagocytosis, but once they entered the blood, tumor cells would be quickly cleared, further inhibiting the growth and proliferation of tumor cells (Zhang R. S. et al., 2021). Another study found that the compound 3-O-{[β-D-glucopyranosyl-(1→2)]-[α-L-rhamnopyranosyl-(1→4)]-β-D-glucopyranosyl} -(25R)-5β-spirostan-3β-ol mainly exerted its effect on inhibiting the proliferation of human large cell lung cancer cells (NCI-H460) by inducing apoptosis and cell cycle arrest, with an IC_50_ value of 1.39 μM (Liu et al., 2021). Besides that, the extract of *A. cochinchinensis* (1–100 mg/ml) dose-dependently not only inhibited the EtOH-induced tumor necrosis TNF-α secretion but also inhibited the EtOH and TNF-α-induced cytotoxicity. In addition, the extract of *A. cochinchinensis* inhibited the TNF-α -induced apoptosis of Hep G2 cells. Therefore, the above results suggest that *A. cochinchinensis* may prevent the EtOH-induced cytotoxicity by inhibiting the apoptosis of Hep G2 cells (Koo et al., 2000). These studies will provide a reference for further in-depth clinical application of *A. cochinchinensis* in cancer treatment.

### Anti-depressant and neuroprotection

The risk of depression has greatly increased due to the enormous mental and physical stress people face due to modern, fast-paced lifestyles (Martins and S., 2018; Angeloni and Vauzour, 2019; Payne et al., 2022). The researchers ovariectomized rats and exposed them to a chronic stress reaction state for 4 weeks. They additionally administered *A. cochinchinensis* extract (1000 and 2000 mg/kg) to observe mental state alterations of the menopausal rats. The results showed that the expression of brain-derived neurotrophic factor (BDNF) and its main receptor tropomyosin receptor kinase B (TrkB) increased in rats. Thus, *A. cochinchinensis* extract could potentially exert anti-depressant effects (Kim et al., 2020). In addition, another study showed that the *A. cochinchinensis* extract, activating phosphatase 2 (Shp-2), ERK1/2, and Akt signaling pathways, could directly affect treating depression and nerve protection (Jalsrai et al., 2016). The pathogenesis of Alzheimer’s disease is unclear, but neuroprotection is shown in different studies to prevent and alleviate it. Lee et al., 2018 study showed that phenols, saponins and protodiosgenin in *A. cochinchinensis* extracts induced enhanced nerve growth factor secretion and decreased intracellular ROS in neurons and microglia cell lines, inhibiting the activity of acetylcholinesterase, thereby improving Alzheimer’s disease (Lee et al., 2018). This study provides novel directions for developing new drugs from *A. cochinchinensis*, and, more importantly, offers new insights into the treatment of Alzheimer’s disease.

### Effects on the gut

Maintaining a normal gut and digestive tract function is one of the key elements to maintaining good health (Sommer et al., 2017; Fassarella et al., 2021; Nathan et al., 2021). Studies have shown that *A. cochinchinensis* extract can treat gut damage caused by metal ions. To evaluate such *A. cochinchinensis* extract effects, the metal ions *Drosophila* model was used. The results showed that *A. cochinchinensis* extract can improved the survival rate of *Drosophila melanogaster*, reduce the mortality of intestinal epithelial cells, and the reduce the intestinal damage caused by metal ions (Zhang L. et al., 2021). At the same time, Kim et al., 2019 found that saponins can increase stool frequency, gastrointestinal transit, mucosal layer thickness, flat luminal surface, and the number of paneth cells, thus playing a role in the treating constipation. Improvements were also observed in the levels of acetylcholine esterase activity, the phosphorylation of myosin light chains, and the expression of muscarinic acetylcholine receptors M2/M3 (Kim et al., 2019). This study provides strong evidence for *A. cochinchinensis* applications in treating certain gut-related diseases. However, another study showed that polysaccharides in *A. cochinchinensis* have a role in gut flora regulation. The impact of inulin-type fructan on gut microbiota was investigated by *in vitro* mediation with human fecal cultures. The results showed that inulin-type fructan was digested by gut microbiota, while the pH value in the *A. cochinchinensis* neutral polysaccharide (ACNP) fecal culture was greatly decreased. The total short-chain fatty acids, acetic, propionic, i-valeric, and n-valeric acids were significantly increased (Sun et al., 2020). Collectively, inulin-type fructan was shown to regulate gut microbiota beneficially (Vandeputte et al., 2017; Tao et al., 2021). Thus, it has the potential to be used as a dietary supplement or drug to improve health.

### Other activities

As *A. cochinchinensis* is widely used as a traditional herbal medicine with high medicinal value, its safety profile is very important. A recent study evaluated the hepatotoxicity and nephrotoxicity of *A. cochinchinensis* toward the livers and kidneys in ICR mice. Female and male ICR mice were orally administered with 150 mg/kg, 300 mg/kg, and 600 mg/kg *A. cochinchinensis* extract for 14 days, respectively, and the changes in relevant markers (organ weight, urine composition, liver pathology, and kidney pathology) were observed. The results showed that Female and male ICR mice were orally administered with 150 mg/kg, 300 mg/kg, and 600 mg/kg *A. cochinchinensis* extract for 14 days, respectively, and the changes in relevant markers (organ weight, urine composition, liver pathology, and kidney pathology) were observed (Sung et al., 2017a). Therefore, the saponins in the *A. cochinchinensis* extract have no specific liver and kidney toxicity, reinforcing the excellent safety profile of *A. cochinchinensis*.

## Applications

**FIGURE 6:**
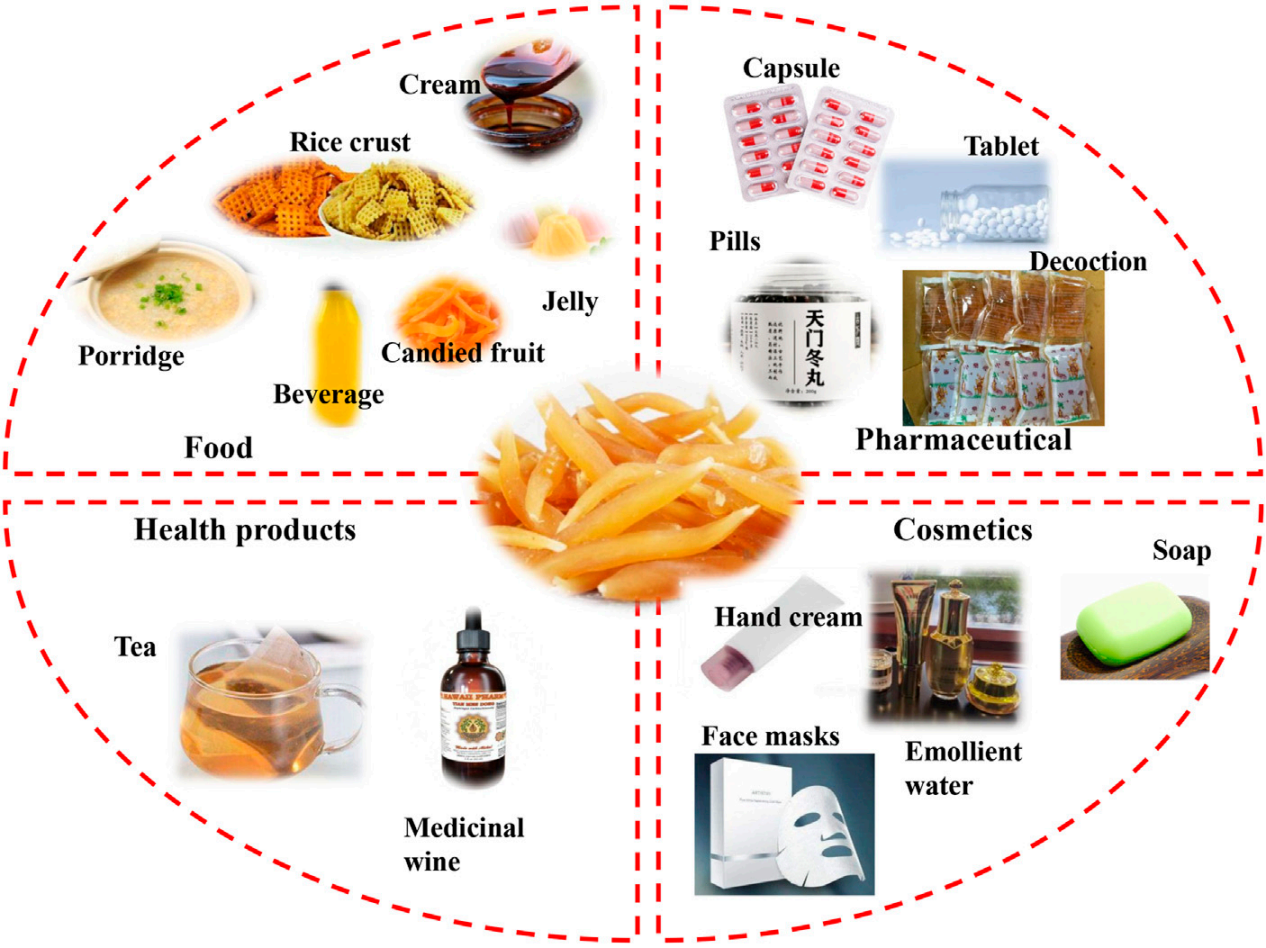
The applications of *A. cochinchinensis* in pharmaceutical, food, health products, and cosmetics.

*A. cochinchinensis* embodies not only significant medicinal value in the field of TCM but also shows distinctive application value in the fields of pharmaceuticals, health care products, food, cosmetics, and others. These applications are summarized in Figure 6, and *A. cochinchinensis* patents in pharmaceuticals, foods, health products and cosmetics are listed in Table 5.

**TABLE 5:** The patents for *A. cochinchinensis*.

NO	Patent name	Approval number
Pharmaceutical		
1	Traditional Chinese medicine pill for treating internal injury cough	CN104013932B
2	A traditional Chinese medicine for treating old cough	CN103611141B
3	Chinese medicine for clearing lung in children	CN103550594B
4	Application and preparation method of pharyngitis tablet	CN103656415B
5	A drug and capsule for constipation	CN103948780B
6	A traditional Chinese medicine preparation for rapid cough relief	CN103028057B
7	A Chinese herbal compound for the treatment of jaundice hepatitis and cholecystitis	CN103349748B
8	A Chinese medicine combination for anti-aging and its preparation method	CN194370700B
9	A pharmaceutical composition for treating juvenile white hair loss	CN103690798B
Food		
10	*Radix asparagi* and *platycodon* grandiflorum healthcare rice crust	CN103652639B
11	*Rehmannia-radix asparagi* beverage and preparation method thereof	CN102150912B
12	A preparation method of health jelly with algae flavor	CN103621855B
Health product		
13	A medicinal wine for lowering blood pressure, blood sugar and blood lipid	CN103860903B
14	A traditional Chinese medicine health wine and its preparation method	CN103013795B
15	A longevity medicinal wine and its preparation method	CN104784474B
16	A health tea for preventing diabetes	CN102935186B
Cosmetic		
17	*A. cochinchinensis* whitening compound soap	CN103361213B
18	Beauty antibacterial soap	CN103589537B
19	A plant combination for delaying skin aging and its preparation method	CN103550511B
20	A Traditional Chinese Medicine Composition for increasing skin moisture and its preparation method	CN101322800B
Others		
21	A compound additive for cigarette and its preparation method and application	CN103549652B
22	A chrysanthemum scented snuff	CN103005679B
23	*A.cochinchinensis* immune adjuvant and influenza vaccine containing the adjuvant	CN101926995B

As mentioned above, *A. cochinchinensis* contains numerous active compounds having many promising effects *in vitro* and *in vivo*, indicating their great potential to for pharmaceutical applications (Ren et al., 2021). The pharmaceutical properties of *A. cochinchinensis* were recorded well in ancient Chinese medical literature. Nowadays, *A. cochinchinensis* has a wide range of clinical applications in the respiratory, digestive, urinary system, with diverse uses. Clinically, *A. cochinchinensis* is often used to treat respiratory diseases such as cough, asthma, and lung cancer. *A. cochinchinensis* can be used alone, in combination with other pharmaceuticals, or for external use (Hong et al., 2000; Liu et al., 2015). Health care products are becoming highly popular as people pay increasing attention to their physical health (Tabatabai and Sellmeyer, 2021). *A. cochinchinensis* through self-fermentation or fermentation with other Chinese herbal medicines, is marketed form of functional medicinal wine (Kim et al., 2017; Wuyts et al., 2020). It contributes to lowering blood pressure, blood sugar, and blood lipid, so it is highly sought after by middle-aged and elderly people (Sikand et al., 2015). Moreover, there are functional teas with health-promoting properties (Fu et al., 2018). *A. cochinchinensis* is also widely used in the food and culinary field. In the folk, people usually use *A. cochinchinensis* is used as the main raw material to cook porridge or paste, used to relieve cough, expectorant, tonsillitis, dry throat, sore throat, hemoptysis, and treat constipation. It is also processed into *A. cochinchinensis* candied fruit, that is popular, especially among young people. Certain modern *A. cochinchinensis* foods products have been patented, such as *Radix asparagi* and *platycodon grandiflorum* healthcare rice crust, *Rehmannia-radix asparagi* beverage and health jelly with algae flavor. Recent studies have shown that long-term consumption of *A. cochinchinensis* as a traditional edible plant can inhibit the production of pro-inflammatory cytokines interleukin-1 beta (IL-1β) and TNF-α, thereby treating various immune-related diseases (Safriani et al., 2022). Despite the currently limited research on *A. cochinchinensis* food products, *A. cochinchinensis* has great potential applications and novel future products in the food field. Interestingly, the extract obtained from fermented *A. cochinchinensis* is also used as a whitening facial mask and whitening soap, with increasing sales, as it can inhibit the formation of tyrosinase and melanin (Sakuma et al., 1999; Pillaiyar et al., 2017). At the same time, patents granted on *A. cochinchinensis* show that it has beneficial properties for improving skin aging, skin whitening, reducing skin wrinkles, and moisturizing, among others. Therefore, the potential of further *A. cochinchinensis* commercial applications in the cosmetics industry should be sought after with increased research efforts.

## Conclusions and perspectives

In this paper, we review the botany, traditional uses, phytochemistry, applications, and pharmacology activities of *A. cochinchinensis* according to ancient classics and modern researches, and it will provide a new insight for future exploration of *A. cochinchinensis.* The root of *A. cochinchinensis* has been widely used to treat cough, fever, pneumonia, stomachache, tracheitis, rhinitis, cataract, acne and urticaria. Meanwhile, the root of *A. cochinchinensis* has a predominant therapeutic effect in diseases such as sthma, constipation, pneumonia. Interestingly, *A. cochinchinensis* exerts multiple functions as medicine, food, and cosmetics, which has been widely used as whitening or healthcare product. Up to now, more than 90 compounds have been isolated and identified from *A. cochinchinensis.* Among these constituents, steroidal saponins represent the main active ingredients. It is expected that more compounds of these categories will be discovered in the future studies. In addition, researches have shown that both extracts and active components of *A. cochinchinensis* possess a wide range of pharmacological activities, including anti-asthma, anti-inflammatory, anti-oxidation, anti-tumor, improving Alzheimer’s disease, nerve protection, gut health-promoting and so on. These modern pharmacological studies supported most traditional uses of *A. cochinchinensis* as an indispensable TCM.

However, gaps still exist in the systematic research on *A. cochinchinensis.* Firstly, reported studies have shown that the main chemical components of *A. cochinchinensis* is steroidal saponins. While other chemical constituents such as polysaccharides, lignans and amino acids extracted and isolated from *A. cochinchinensis* are very few compared with steroidal saponins. More chemical constituents must be obtained to explore the relationship between compounds and pharmacological effects in depth. Therefore, new separation and analysis techniques should be developed and implemented to analyze and determine *A. cochinchinensis* chemical composition comprehensively. Secondly, quality standards have not been adequately set. Since *A. cochinchinensis* has a wide variety and is easily confused with other varieties, it is very necessary to establish a complete set of quality standards to distinguish these products. This will also contribute to better-protecting people’s health and safety. Thus, it is crucial to establish the *A. cochinchinensis* quality analytical standards and find the appropriate markers to implement such quality control. At the same time, it is also necessary to conduct systematic and in-depth research on the toxicology of *A. cochinchinensis* to improve the safety profile of its clinical use. Thirdly, the main part of *A. cochinchinensis* used for medicinal compound extraction is its dried root, and the other parts are discarded. However, the resources of roots are relatively rare compared to the resources of leaves and fruits. In the future, in-depth research should be conducted on the leaves and fruits of *A. cochinchinensis*, to explore their value so that the plant can be fully utilized. This reduces the waste of plant resources and might contribute to the development of new drugs, as novel compounds might be discovered in other plant parts. Therefore, we should solve the existing problems as soon as possible, so that the future development of *A. cochinchinensis* will be better.

In addition, in order to further elucidate the mechanism of *A. cochinchinensis* in treating diseases, it is essential to establish the internal relationship between chemical components and their pharmacological activities. Pharmacokinetic studies of *A. cochinchinensis* can also be conducted to try to elucidate its changes including absorption, distribution, metabolism and excretion. This will further elucidate the complex relationship between chemical components and clinical effects to reveal potential mechanism of action. At the same time, *A. cochinchinensis* can also be used as food and nutritional supplement. People become more aware of their health, edible Chinese herbal medicines with health-promoting and therapeutic effects are becoming very popular. On this basis, in-depth research should be conducted in the fields of *A. cochinchinensis* health products, food, and cosmetics, which may have broader prospects for future development, providing new idea for *A. cochinchinensis* research.

To sum up, the root of *A. cochinchinensis* is an important edible medicinal herb with extensive pharmacological activities and great values in medicine, food, and cosmetics. However, more in-depth and comprehensive studies on clinical utility are needed to determine its safety and availability. Until now, multiple compounds have been discovered in *A. cochinchinensis*, but what we have done is far from enough. Furthermore, the precise molecular mechanisms of these active ingredients in some diseases still worth further study. Consequently, systematic studies on phytochemistry and bioactivities of *A. cochinchinensis* will undoubtedly be the key direction of future research. This review should provide an important reference for the development and application of *A. cochinchinensis*. Siand et al., 2015, Sheng, 2022b.

## Glossary

ACNP: *Asparagus cochinchinensis* neutral polysaccharide
ACNVs: *Asparagus cochinchinensis*-derived nanovesicles
Ara: Arabinose
BDNF: Brain-derived neurotrophic factor
CAT: Catalase
COX-2: Cyclooxygenase-2
CZE: Capillary zone electrophoresis
DPPH: 1,1-diphenyl-2-picrylhydrazyl radical 2,2-diphenyl-1-(2,4,6-trinitrophenyl) hydrazyl
Fru: Fructose
Gal: Galactose
GalUA: Galacturonic acid
Glc: Glucose
GlcUA: Glucuronic acid
GPX: Glutathione peroxidase
Ig E: Immunoglobulin E
IL-4: Interleukin 4
IL-13: Interleukin 13
IL-1β: Interleukin-1 beta
iNOS: Inducible nitric oxide synthase
LPS: Lipopolysaccharide
Man: Mannose
MDA: Malondialdehyde
MPO: Myeloperoxidase
NGF: Nerve growth factor
NO: Nitric oxide
NOS: Nitric oxide synthase
OVA: Ovalbumin
PA: Phthalic anhydride
ROS: Reactive oxygen species
Rha: Rhamnose
SOD: Superoxide dismutase
SP: Substance P
TCM: Traditional Chinese medicines
Tg: Transgenic
TNF-α: Tumor necrosis factor-α
TPA: 12-O-tetradecanoyl-phorbol-13-acetate
TrkB: Tropomyosin receptor kinase B
Xyl: Xylose
